# A new function for drug combination dose finding trials

**DOI:** 10.1038/s41598-024-53155-4

**Published:** 2024-02-12

**Authors:** Jiacheng Xiao, Weijia Zhang

**Affiliations:** Department of Financial and Actuarial Mathematics, Xi’an Jiaotong-Liverpool University, Suzhou, 215123 Jiangsu China

**Keywords:** Statistics, Clinical trials

## Abstract

Combination drugs play an essential role in treating cancers. The challenging part of the combination drugs are to specify the dose-toxicity ordering, which means the sequences of dose escalation and de-escalation in process of dose findings should be pre-determined. In the paper, we extend a novel function of the continual reassessment method based on the combination of the normal distribution for drug-combination dose-finding trials and systematically evaluate its performance using a template of four performance measures EARS (Efficiency, Accuracy, Reliability, Selection). Dose escalation and deescalation rules are based on the nearest neighborhood continual reassessment method for a combination drug, and we specify all possible dose-toxicity orderings in the trial. Simulation demonstrates that the new design is efficient, accurate and reasonably reliable.

## Introduction

Phase I dose-finding clinical trials are crucial for the overall success of new drug development. The dose-finding trial is the first stage in the human clinical trial and forms the foundation for the proof-of-concept Phase II and confirmatory Phase III clinical trials. Its goal is to reliably identify the maximum tolerated dose (MTD) with a high probability while minimizing the use of unsafe and ineffective doses in trial patients. The MTD and the dose escalation rule are often determined with a target dose-limiting toxicity (DLT) probability. Specifically, the MTD is defined as a dose level whose DLT probability is closest to a given target toxicity rate.

As experiments on human subjects, phase I dose-finding trials typically face the trade-off between exploration or collective ethics (i.e., identifying the efficacious dose for the overall success of the entire clinical trial) and exploitation or individual ethics (i.e., avoiding unsafe or ineffective treatments to trial subjects). Moreover, any statistical design of phase I clinical trials is fundamentally adaptive because dose escalation and de-escalation decisions depend on previous dose selections and toxicity responses. There are typically three types of dose escalation methods: rule-based (or algorithm-based, nonparametric) designs, model-based (or parametric) designs, and model-assisted designs. Rule-based methods include the 3+3 (or up-and-down) design^1^ and its variants^2^. Although they are easy to implement and popular in practice, they are nevertheless suboptimal from both ethical and statistical perspectives^3^.

The continual reassessment method (CRM) by O’Quigley et al.^4^ is a popular model-based design of phase I clinical trials. It is based on the idea of modeling the true toxicity probability curve by a parametric probability model with unknown parameters, which are estimated by either the Bayesian or frequentist approach. Given current information and updated estimates of toxicity probabilities at all dose levels, the next patient (or cohort of patients) is assigned to the dose level whose estimated toxicity probability is closest to the given target toxicity rate. Many statistical properties, practical issues and methodological extensions of CRM for only one drug have been investigated and published in the literature^5–9^. The researchers comprehensively review various types of nonparametric, parametric and semi-parametric designs, and discuss about important issues such as overdose control, Bayesian decision theoretical designs and other optimal designs, and their data analysis^10–14^.

In recent years, significant interest and progress in personalized medicine, molecularly targeted therapies, and drug combinations have been developed rapidly. For cytotoxic treatment of cancer, one particular drug may show effectiveness to destroy the cancerous cell, but cellular heterogeneity may create a certain drug-resistant disease^15,16^. Because drug susceptibility varies among cells and between patients, a combination of drugs can help achieve the desired treatment intensity and resistance, when the drugs have non-overlapping toxicities and the dose combination is not overly toxic^17^. For example, drug combinations are shown to be effective in enhancing survival in early and advanced stage cancer patients, and even curative in testicular cancer patients^18^.

When a combination of drugs is used to treat cancer, it is important to determine the maximum tolerated dose combination (MTD combination) of all drugs involved. This is not a simple issue because the toxicity probability model of dose combination may be significantly influenced by potential pharmacokinetic and pharmacodynamic interactions between the drugs^16,19,20^. When the continual reassessment method is applied for a single cytotoxic drug, an important assumption is that the toxicity probability is monotonically increasing in the drug dose level. That is, a higher dose corresponds to a higher probability of toxicity as well as a higher probability of efficacy. However, this simple order fails for the toxicity probability of drug dose combinations. The monotonicity assumption is true only partially for a subset of dose combination levels, for example, when the dose level increases in one cytotoxic agent but the dose level for the other drug is kept constant.

The idea of incorporating the prior information based on the single drug toxicity profiles with CRM^21,22^ is like dimension reduction. Wages et al.^23,24^use CRM to escalate or deescalate the drug combination based on possible partial orders and discuss the issues of choosing partial orders for the combination drugs when the power model is used with CRM. Two stages Bayesian method for drug combination escalation, using the diagonal movement in the first stage and toxicity equivalence contour in the second stage^25^ and the Bayesian adaptive design based on robust dimension reduction is also applied for CRM^13,26,27^, and then the performance of different drug combination dose-finding trials are compared, including the up-and-down designs, CRM with partial ordering, copula regression and the latent contingency table^28,29^. Sweeting et al.^30^ investigates the performance of different escalation strategies for phase I drug combination trials, and shows that strategies allowing only non-diagonal escalations are inefficient and identify the fewer maximum tolerated dose combinations. Further improvements and model misspecification of these methods are discussed^17,31,32^ . On the other hand, copulas are used to describe stochastic dependence in phase I drug combination trials^33^, and logistic regression with covariates is applied for the Bayesian design of phase I trial for combination drugs^34^. The risk of overdose for combination drugs is still a big issue as that for a single agent^25,35^. Finally, some reviews are proposed to discuss different issues and approaches for drug combination phase I clinical trials^9,30,36^.

Although model-based parametric designs such as CRM are efficient in identifying the MTD, such designs suffer from some limitations such as the choice of the parametric model, the sequential update of parameters of the model, the robustness of the model, and the universal superiority of a model in all possible scenarios. Model-assisted designs are introduced to take advantage of both rule-based and model-based designs, such as the Bayesian Optimal Interval Design or BOIN^19^ and the keyboard design^37^. Later, these are extended to drug combination trials^13,38^.

In this paper, we extend the dose toxicity probability function^39^ to drug combinations and compare its performance against the power and hyperbolic tangent functions. Dose escalation is an extension of the up-and-down design. After each observation, dose escalation or deescalation occurs within the nearest rectangular neighborhood are introduced^28,40^. The advantage of this method is that the new function of the CRM design can change $\sigma ^2$ of the cumulative distribution function of the normal distribution in our model to adjust the shape of the dose-toxicity probability curve according to our demands. To generate a class of different shapes, the different demands of relationship between dose level and toxicity probability can be achieved. For example, at the lower, in the middle and at the higher dose stages of the trial, it is required to be in smooth, steep and smooth shape of curve, respectively. Secondly, for combination drugs, it is more difficult to determine that the toxicity probability monotonically increasing than by the dose level for a single drug, and the ordering of toxicity probabilities of dose combination is unknown. In the other words, for drug combination, the direction of dose escalation and deescalation is uncertain. But we can determine this order anthropologically in advance. For instance, for 3 by 3 combination drug dose levels, the toxicity probability of dose combination (3, 2) is greater than that of dose combination (2, 2), meanwhile, we know the toxicity probability of dose combination (2, 3) is greater than that of dose combination (2, 2). But we may not know whether the toxicity probability of dose combination (3, 2) is greater than that of dose combination (2, 3). So specifying a possible dose-toxicity ordering is a primary goal of CRM with combination drugs. The motivation of this paper is that we propose to acquire a predetermined dose escalation-deescalation scheme in which way we could be able to solve the issue in the similar way for a single agent.

This paper is organized as follows. In section “Continual reassessment method for drug combinations, the toxicity probability function of CRM^39^ is extended to drug combinations. Section “Simulation studies” introduces the setting of simulation and scenarios. A comprehensive comparison of the new toxicity probability function with the power and hyperbolic tangent functions is given in section “Comparison of different CRM functions”. Section “Conclusion” concludes the paper.

## Continual reassessment method for drug combinations

Consider a phase I clinical trial in which patients are treated sequentially, one at a time, with a combination of two drugs *A* and *B*. Drug *A* has *K* dose levels and drug *B* has *L* dose levels. Using the CRM function for only one drug^39,41^, we assume that the true toxicity probability at dose combination $d_{i,j}$, $i=1,2, \ldots ,K$ and $j=1,2, \ldots , L$, is given by a parametric function

$$\begin{aligned} P({\text{ toxicity at dose combination }}\; d_{i,j})=\pi _{i,j}= \frac{2\Phi (\beta +\alpha p_{i,j})}{1+\Phi (\beta +\alpha p_{i,j})}, \end{aligned}$$

where $\Phi$ is the cumulative distribution function of the normal distribution $N(0, \sigma ^2)$, $\sigma ^2$ is unknown and follows a prior distribution. However, $\beta$ and $\alpha >0$ are given constants. Moreover, $\{p_{i,j}, i=1,2, \ldots ,K, j=1,2, \ldots , L\}$ are pre-specified skeleton probabilities.

To safeguard individual ethics of trial subjects, an ethically acceptable target DLT rate $\theta \in (0, 1)$ is specified (such as 0.3 in this paper). The goal is to reliably identify the maximum tolerated dose combination $(i^*, j^*)$ with a high probability, with minimal application of unsafe and ineffective dose combinations. The maximum tolerated dose combination $(i^*, j^*)$ is defined as the dose combination levels whose final estimated toxicity probability is nearest $\theta$. That is, $(i^*, j^*) = \arg \min _{\{(i, j), i=1,2, \ldots ,K, j=1,2, \ldots , L\}}\{|\hat{\pi }_{i,j} - \theta |\}.$

**Table 1:** Drugs A and B dose combinations, 10 scenarios of true toxicity probabilities, and common skeletons. Underline indicates the target DLT rate.

Doses of A	B				Scenario 1				Scenario 2			
	1	2	3	4	1	2	3	4	1	2	3	4
1	$d_{11}$	$d_{12}$	$d_{13}$	$d_{14}$	0.3	0.4	0.5	0.55	0.15	0.3	0.35	0.4
2	$d_{21}$	$d_{22}$	$d_{23}$	$d_{24}$	0.4	0.55	0.6	0.65	0.3	0.35	0.42	0.5
3	$d_{31}$	$d_{32}$	$d_{33}$	$d_{34}$	0.5	0.6	0.65	0.75	0.38	0.4	0.5	0.6
4	$d_{41}$	$d_{42}$	$d_{43}$	$d_{44}$	0.6	0.7	0.75	0.8	0.42	0.5	0.6	0.7
5	$d_{51}$	$d_{52}$	$d_{53}$	$d_{54}$	0.7	0.78	0.85	0.9	0.5	0.6	0.7	0.8

Doses of A	Scenario 3				Scenario 4				Scenario 5			
	1	2	3	4	1	2	3	4	1	2	3	4
1	0.1	0.2	0.3	0.4	0.05	0.1	0.2	0.3	0.05	0.1	0.15	0.2
2	0.2	0.3	0.4	0.5	0.15	0.25	0.3	0.35	0.1	0.15	0.2	0.25
3	0.3	0.4	0.5	0.6	0.25	0.3	0.4	0.5	0.15	0.2	0.3	0.4
4	0.4	0.5	0.6	0.7	0.3	0.35	0.45	0.55	0.2	0.3	0.4	0.5
5	0.5	0.6	0.7	0.8	0.35	0.4	0.5	0.6	0.4	0.5	0.6	0.7

Doses of A	Scenario 6				Scenario 7				Scenario 8			
	1	2	3	4	1	2	3	4	1	2	3	4
1	0.05	0.1	0.15	0.2	0.02	0.05	0.08	0.1	0.02	0.05	0.08	0.1
2	0.1	0.15	0.2	0.25	0.05	0.08	0.1	0.15	0.03	0.06	0.1	0.15
3	0.15	0.2	0.25	0.3	0.1	0.15	0.2	0.25	0.09	0.12	0.18	0.2
4	0.2	0.25	0.3	0.35	0.15	0.2	0.25	0.3	0.15	0.18	0.2	0.25
5	0.25	0.3	0.4	0.5	0.2	0.25	0.3	0.4	0.18	0.2	0.25	0.3

Doses of A	Scenario 9				Scenario 10				Common skeleton values			
	1	2	3	4	1	2	3	4	1	2	3	4
1	0.05	0.1	0.15	0.25	0.1	0.2	0.35	0.4	0.0211	0.0381	0.0629	0.0961
2	0.1	0.15	0.25	0.35	0.15	0.3	0.4	0.45	0.1376	0.1865	0.2413	0.3
3	0.2	0.25	0.3	0.4	0.2	0.4	0.45	0.5	0.3608	0.4218	0.4815	0.5385
4	0.3	0.35	0.4	0.5	0.25	0.45	0.55	0.65	0.5921	0.6416	0.6868	0.7275
5	0.35	0.45	0.5	0.6	0.3	0.6	0.7	0.8	0.7639	0.7961	0.8244	0.8491

## Simulation studies

By means of simulation, we compare the performance of our new CRM function against the power and hyperbolic tangent functions. To be more specific, the following CRM functions are used in all simulations:

- the new function $\pi _{i,j}= \frac{2\Phi (\beta +\alpha p_{i,j})}{1+\Phi (\beta +\alpha p_{i,j})},$ where $\Phi$ is the cumulative distribution function of the normal $N(0, \sigma ^2)$ distribution, $\beta = -5$ and $\alpha =4$, and $1/\sigma ^2$ follows a prior distribution;
- the power function $\pi _{i,j} = p_{ij}^{\alpha }$, where $\alpha$ follows a prior distribution;
- the hyperbolic tangent function, $\pi _{i,j}= \left\{ \frac{\left( e^{2p_{ij}}-1\right) /\left( e^{2p_{ij}}+1\right) +1}{2}\right\} ^{\alpha },$ where $\alpha$ follows a prior distribution;
- for all models, the prior distribution for the unknown parameter is the *gamma*(*x*, 0.5, 0.5) distribution. It has been used in previous literature^41^.

Moreover, $\{p_{i,j}, i=1,2, \ldots ,K, j=1,2, \ldots , L\}$ are pre-specified skeleton values that are used for all functions and all simulation scenarios, in order to understand the robustness of these CRM functions.

We take $K=5$ and $L=4$, and simulate 10 different scenarios of true toxicity probabilities which are listed in Table 1, together with a list of skeleton values that is used for all scenarios. In all scenarios, the target DLT rate is $\theta = 0.3$. Each scenario is simulated 1000 times. The number of patients in each trial is 30.

**Table 2:** Simulated values of the criteria EAR. A good design should produce small efficiency values, large accuracy values, and low reliability values. Underline indicates the best value in each scenario.

Scenario	Model	Efficiency		Accuracy		Reliability				
		E1	E2	A1	A2	R1	R2	R3	R4	R5
1	New	0	0	0.794	0.742	0	0.013	0.206	0.258	0.359
	Power	0	0	0.679	0.578	0.051	0.18	0.321	0.422	0.374
	Hyperbolic tangent	0	0	0.773	0.739	0	0.024	0.227	0.261	0.346
2	New	0.289	0.357	0.09	0.066	0.001	0.922	0.531	0.511	0.319
	Power	0.123	0.197	0.156	0.117	0.109	0.712	0.564	0.57	0.329
	Hyperbolic tangent	0.253	0.351	0.09	0.086	0.005	0.812	0.567	0.477	0.299
3	New	0.294	0.356	0.094	0.07	0.001	0.878	0.425	0.435	0.318
	Power	0.244	0.288	0.137	0.106	0.07	0.745	0.345	0.393	0.317
	Hyperbolic tangent	0.26	0.38	0.115	0.079	0.005	0.797	0.394	0.384	0.293
4	New	0.275	0.372	0.086	0.052	0.003	0.878	0.383	0.421	0.306
	Power	0.322	0.4	0.099	0.081	0.073	0.806	0.281	0.278	0.285
	Hyperbolic tangent	0.326	0.438	0.096	0.07	0.024	0.795	0.291	0.281	0.267
5	New	0.41	0.45	0.151	0.106	0.006	0.683	0.287	0.337	0.292
	Power	0.528	0.61	0.141	0.096	0.051	0.748	0.191	0.198	0.249
	Hyperbolic tangent	0.545	0.647	0.13	0.09	0.011	0.741	0.194	0.173	0.233
6	New	0.498	0.505	0.1	0.075	0.044	0.8	0.203	0.272	0.275
	Power	0.664	0.718	0.08	0.065	0.043	0.845	0.096	0.088	0.229
	Hyperbolic tangent	0.659	0.752	0.079	0.062	0.001	0.837	0.104	0.062	0.216
7	New	0.551	0.562	0.141	0.107	0.175	0.699	0.166	0.223	0.257
	Power	0.775	0.829	0.099	0.081	0	0.811	0.028	0.009	0.2
	Hyperbolic tangent	0.742	0.867	0.091	0.049	0.01	0.864	0.076	0.035	0.19
8	New	0.586	0.619	0.414	0.381	0	0.392	0	0	0.226
	Power	0.935	0.975	0.065	0.025	0	0.942	0	0	0.179
	Hyperbolic tangent	0.812	0.923	0.188	0.077	0	0.799	0	0	0.17
9	New	0.336	0.393	0.119	0.085	0.013	0.772	0.425	0.438	0.301
	Power	0.455	0.533	0.093	0.073	0.114	0.828	0.359	0.321	0.268
	Hyperbolic tangent	0.489	0.582	0.088	0.067	0.034	0.816	0.335	0.284	0.25
10	New	0.349	0.387	0.058	0.055	0	0.899	0.536	0.503	0.314
	Power	0.334	0.368	0.085	0.07	0.088	0.842	0.496	0.492	0.309
	Hyperbolic tangent	0.318	0.419	0.061	0.051	0.01	0.874	0.56	0.478	0.287

Let $(a_i, b_i)$ be the dose combination for patient $i, i=1, 2, \ldots , 30$, $a_i=1, \ldots , K$, $b_i = 1, \ldots , L$, and $y_i$ be the toxicity response from patient *i*. For each chosen scenario, the NNCRM method follows these steps:

- Step 1. The first patient is always treated at the lowest drug combination dose level. Set $(a_1, b_1)=(1, 1)$, simulate toxicity response and record its observation $y_1$.
- Step 2. Suppose we now treat the *j*th patient, where $j=2, \ldots , 30.$ Assume that we have observed information $D_j=\{ (a_1, b_1, y_1), \ldots , (a_{j-1}, b_{j-1}, y_{j-1})\}$ from previously treated patients. Based on $D_j$, update the likelihood function
  $$\begin{aligned} L(X|D_j)=\prod _{k=1}^{j-1} \pi _{a_k,b_k}^{y_k} (1-\pi _{a_k,b_k})^{1-y_k} \end{aligned}$$
  and calculate the posterior mean toxicity probabilities
  $$\begin{aligned} \hat{\pi }_{a, b}=\int \pi _{a,b} \frac{L(X|D_j)f(X)}{\int L(X|D_j)f(X)d(X)}d(X) \end{aligned}$$
  at all dose combination $d_{a,b}=(a, b)$, $a=1, \ldots , K, b = 1, \ldots , L,$ where $K=5, L=4$, and $X=1/\sigma ^2$ for our new function and $X=\alpha$ for the power and hyperbolic tangent functions, and $f(\cdot )$ is the prior distribution of *X*.
- Step 3. Within the nearest (rectangular) neighborhood $\{ (a, b): \max (1, a_{j-1}-1)\le a\le \min (a_{j-1}+1, K), \max (1, b_{j-1}-1)\le b\le \min (b_{j-1}+1, L)\}$, determine the dose combination $(a_j, b_j) = \arg \min _{(a, b)} \{|\hat{\pi }_{a, b} - \theta |\}$, at which the posterior mean toxicity probability is nearest the target DLT rate of $\theta =0.3$.
- Step 4. For the *j*th patient, escalate or deescalate to the dose combination $(a_j, b_j)$. Simulate and record toxicity response $y_j$ and add $(a_j, b_j, y_j)$ to the list of all observations.
- Step 5. Repeat Steps 2 to 4 until all trial patients (i.e., 30) are completed.

## Comparison of different CRM functions

We summarize simulation results and compare the performance of different CRM functions using the evaluation criteria EARS: Efficiency, Accuracy, Reliability, Selection.

**Table 3:** Simulated true MTD combination selection. Underline indicates the best design in each scenario.

Scenario	New function				Power function				Hyperbolic tangent function			
1	0.794	0.07	0.046	0.019	0.679	0.174	0.066	0.014	0.773	0.093	0.076	0.036
	0.028	0.028	0.011	0.003	0.047	0.01	0.003	0	0.005	0.01	0.006	0.001
	0	0.001	0	0	0.006	0.001	0	0	0	0	0	0
	0	0	0	0	0	0	0	0	0	0	0	0
	0	0	0	0	0	0	0	0	0	0	0	0
2	0.242	0.132	0.061	0.05	0.123	0.197	0.165	0.083	0.253	0.113	0.148	0.153
	0.099	0.19	0.162	0.071	0.116	0.106	0.086	0.015	0.067	0.086	0.079	0.035
	0.038	0.054	0.072	0.028	0.039	0.046	0.013	0.005	0.032	0.02	0.01	0.002
	0.005	0.008	0.016	0.014	0.006	0	0	0	0.002	0	0	0
	0.001	0	0	0	0	0	0	0	0	0	0	0
3	0.146	0.089	0.09	0.073	0.016	0.126	0.163	0.088	0.089	0.090	0.163	0.144
	0.059	0.137	0.145	0.092	0.102	0.181	0.117	0.037	0.081	0.137	0.142	0.047
	0.054	0.053	0.041	0.009	0.067	0.071	0.016	0.007	0.046	0.037	0.018	0.003
	0.007	0.004	0	0.001	0.009	0	0	0	0.003	0	0	0
	0	0	0	0	0	0	0	0	0	0	0	0
4	0.024	0.029	0.036	0.043	0	0.007	0.058	0.109	0.008	0.023	0.05	0.086
	0.027	0.087	0.124	0.135	0.042	0.13	0.142	0.12	0.044	0.116	0.17	0.156
	0.072	0.132	0.124	0.047	0.085	0.105	0.075	0.02	0.085	0.103	0.081	0.029
	0.043	0.037	0.018	0.007	0.041	0.043	0.016	0.005	0.024	0.011	0.011	0
	0.007	0.004	0.004	0	0.002	0	0	0	0	0.001	0.002	0
5	0.007	0.003	0.006	0.006	0	0	0.013	0.048	0.003	0.002	0.003	0.02
	0.006	0.018	0.062	0.083	0.006	0.031	0.066	0.137	0.003	0.028	0.079	0.151
	0.042	0.112	0.199	0.148	0.03	0.135	0.18	0.094	0.043	0.165	0.211	0.127
	0.065	0.104	0.082	0.04	0.062	0.101	0.069	0.023	0.048	0.05	0.042	0.018
	0.008	0.004	0.005	0	0.005	0	0	0	0.005	0	0.002	0
6	0.003	0	0.003	0.001	0	0	0.013	0.048	0.003	0.002	0.003	0.02
	0.005	0.009	0.028	0.054	0.006	0.031	0.062	0.133	0.002	0.027	0.071	0.14
	0.027	0.051	0.108	0.123	0.021	0.082	0.114	0.134	0.025	0.093	0.139	0.151
	0.07	0.092	0.126	0.104	0.049	0.085	0.085	0.079	0.056	0.062	0.077	0.062
	0.047	0.05	0.072	0.027	0.02	0.021	0.012	0.005	0.016	0.009	0.028	0.014
7	0	0	0	0.001	0	0	0	0.003	0	0	0	0.001
	0	0	0.003	0.013	0	0.003	0.009	0.05	0.001	0.001	0.004	0.035
	0.006	0.016	0.036	0.085	0.006	0.069	0.101	0.18	0.011	0.049	0.125	0.198
	0.041	0.088	0.118	0.142	0.024	0.097	0.158	0.165	0.051	0.096	0.120	0.125
	0.069	0.075	0.141	0.166	0.029	0.046	0.032	0.028	0.022	0.028	0.057	0.076
8	0	0	0	0	0	0	0	0.001	0	0	0	0
	0	0	0.002	0.006	0	0.002	0.009	0.038	0	0.001	0.003	0.029
	0.002	0.007	0.015	0.055	0.002	0.034	0.077	0.144	0.002	0.033	0.053	0.158
	0.01	0.054	0.079	0.097	0.018	0.078	0.14	0.232	0.036	0.097	0.125	0.131
	0.041	0.069	0.149	0.414	0.026	0.08	0.054	0.065	0.025	0.039	0.08	0.188
9	0.007	0.011	0.013	0.023	0	0	0.023	0.064	0.005	0.004	0.008	0.04
	0.008	0.042	0.085	0.108	0.006	0.064	0.139	0.155	0.015	0.066	0.154	0.161
	0.048	0.099	0.162	0.126	0.052	0.107	0.13	0.081	0.07	0.127	0.133	0.085
	0.077	0.062	0.061	0.031	0.056	0.064	0.042	0.01	0.043	0.036	0.029	0.014
	0.02	0.007	0.005	0.005	0.005	0.001	0.001	0	0.004	0.003	0.003	0
10	0.129	0.079	0.084	0.055	0.019	0.127	0.142	0.059	0.093	0.091	0.133	0.124
	0.046	0.115	0.126	0.099	0.08	0.17	0.093	0.043	0.073	0.122	0.121	0.055
	0.064	0.076	0.052	0.022	0.079	0.091	0.04	0.008	0.05	0.07	0.032	0.014
	0.031	0.017	0.004	0.001	0.029	0.017	0.003	0	0.011	0.009	0.002	0
	0	0	0	0	0	0	0	0	0	0	0	0

- **Efficiency**^42^.
  - Criterion: The efficiency criterion is determined by the observed percentage of patients allocated to sub-therapeutic dose combinations whose toxicity probabilities are less than the target DLT rate.
    - The efficiency measure **E1** gives the proportion of the simulation runs that incorrectly identify sub-therapeutic dose combinations as the true MTD combination. The lower the proportion E1, the lower is the probability for the design to assign patients to ineffective dose combinations.
    - The efficiency measure **E2** calculates the percentage of patients assigned to sub-therapeutic dose combinations. The lower the mean, the lower is the proportion of patients assigned to ineffective dose combinations.
  - Results: From Table 2, our new function is most efficient in 6 out of 10 scenarios.
- **Accuracy**^42^.
  - Criterion: The accuracy measure is concerned with the allocation of patients to the true MTD combinations. In the case of multiple true MTD combinations, we report the average value per true MTD combination.
    - The criterion **A1** calculates the proportion of simulation runs that identify the true MTD combinations correctly. The higher this proportion, the more accurate is the design. ? also name this the percentage of correct selection (PCS).
    - The criterion **A2** derives the mean percentage of patients allocated to the true MTD combinations. The greater the mean, the better is the design.
  - Results: From Table 2, our new function is most accurate in 6 out of 10 scenarios.
- **Reliability**^42^.
  - Criterion: The reliability criterion measures the risk of overdosing.
    - The criterion **R1** reports the proportion of simulation runs that allocate more than 50% of patients to a dose higher than the true MTD combination. The lower the measure, the less is the proportion of patients assigned to unsafe dose combinations.
    - The criterion **R2** reports the proportion of simulation runs with less than one-sixth (i.e., 5 out of 30) of patients allocated per true MTD combination. The lower the proportion, the more reliable is the design.
    - The criterion **R3** calculates the proportion of simulation runs that incorrectly identify unsafe dose combinations (i.e., dose combinations whose toxicity probabilities are higher than the target DLT rate) as the true MTD combination. The lower its value, the lower is the probability of applying an unsafe dose combination ?.
    - The criterion **R4** gives the mean percentage of patients allocated to unsafe dose combinations whose toxicity probabilities are higher than the target DLT rate. The lower the mean, the safer is to avoid the exposure of unsafe dose combinations.
    - The criterion **R5** is the mean percentage (out of the total of 30 patients) of observed DLT’s over all simulation runs, at all dose combinations. The lower the mean percentage, the safer is the design. This measure is used in Ref.^43^.
  - Results: From Table 2, our new function has the lowest value of **R1** in 7 out of 10 scenarios and the lowest value of **R2** in 6 out of 10 scenarios. Although our new function dose not offer the lowest values of **R3**, **R4** and **R5** in some scenarios, its values are not excessively large. Combining all reliability measures, our new function is overall reliable.
- **Selection**.
  - Criterion: Table 3 gives the observed proportions of final true MTD combination selections, out of 1000 simulation runs. A CRM design with a higher proportion of correct true MTD combination selection is better.
  - Results: Our new function is the best overall (with the largest value of the total proportion at all true MTD combinations in scenarios 1, 5, 6, 7, 8, 9).

## Conclusion

We have applied our previously introduced CRM function for a single drug to a combination of two drugs, and compared its performance against the power and hyperbolic tangent functions by means of simulation. Results have demonstrated that our new function has very good behaviors regarding the Sample mean, efficiency, accuracy and reliability, and final correct selection of the true MTD combinations. We have modified slightly our previous term of BEARS by combining Safety with Reliability and adding Selection.

There are several significant and improved differences between this and our past work. Firstly, since we do not know in advance the true toxicity probabilities, we have applied the same list of skeleton values for all possible scenarios of true toxicity probabilities. This helps establish the robustness of the CRM design. Secondly, although by means of simulation we have observed better performance of our new function by choosing different values of $\alpha$ and $\beta$ for different scenarios, we pick only one set of values $\beta = -5$ and $\alpha =4$ in all simulations. This avoids cherry-picking and also assesses the robustness of the new CRM function. Lastly, in our previous publication ?, the CRM design for a single drug is good in the sense that no patient is allocated to unsafe doses. However, the drawback is that no toxicity information is available on high dose levels and it is not reliable to perform statistical analysis of the true MTD. For drug combination, we have avoided this pitfall by tuning the parameters of our new CRM function so that it allocates patients to virtually all possible dose combinations.

We have studied the performance of three CRM functions: our new function, and the power and hyperbolic tangent functions. Both the power and hyperbolic tangent functions are parsimonious with only one parameter $\alpha$, which is treated to be random under the Bayesian approach. They may be regarded as fixed designs because there are no tuning parameters. On the other hand, our new function is more complex with few tuning parameters. But on the other side of the same coin, having tuning parameters gives us some flexibility and possible improvement, and if the right values are chosen for the tuning parameters, our new function can actually do better than the fixed designs.

In fact, by trial and error, we have identified different values of $\alpha$ and $\beta$ for any chosen scenario such that our new function actually performs better than the fixed designs. However, no set of $\alpha$ and $\beta$ values is the best in all scenarios. To avoid cherry-picking and maintain uniformity, we decide to report only one common set of values for $\alpha$ and $\beta$ which give reasonably good results in all scenarios.

## Data Availability

The datasets generated and analyzed during the current study are not publicly available due to the data is simulated by code but are available from the corresponding author on reasonable request.
